# Overnight declarative memory consolidation and non-rapid eye movement sleep electroencephalographic oscillations in older adults with obstructive sleep apnea

**DOI:** 10.1093/sleep/zsad087

**Published:** 2023-04-13

**Authors:** Jun Z Teh, Lucinda Grummitt, Carla Haroutonian, Nathan E Cross, Bradley Skinner, Delwyn J Bartlett, Brendon Yee, Ronald R Grunstein, Sharon L Naismith, Angela L D’Rozario

**Affiliations:** School of Psychology, Faculty of Science, Brain and Mind Centre and Charles Perkins Centre, University of Sydney, Sydney, NSW, Australia; CIRUS Centre for Sleep and Chronobiology, Woolcock Institute of Medical Research, Macquarie University, Sydney, NSW, Australia; NHMRC Centre of Research Excellence to Optimise Sleep in Brain Ageing and Neurodegeneration (CogSleep CRE), Sydney, NSW, Australia; School of Psychology, Faculty of Science, Brain and Mind Centre and Charles Perkins Centre, University of Sydney, Sydney, NSW, Australia; School of Psychology, Faculty of Science, Brain and Mind Centre and Charles Perkins Centre, University of Sydney, Sydney, NSW, Australia; CIRUS Centre for Sleep and Chronobiology, Woolcock Institute of Medical Research, Macquarie University, Sydney, NSW, Australia; CIRUS Centre for Sleep and Chronobiology, Woolcock Institute of Medical Research, Macquarie University, Sydney, NSW, Australia; CIRUS Centre for Sleep and Chronobiology, Woolcock Institute of Medical Research, Macquarie University, Sydney, NSW, Australia; CIRUS Centre for Sleep and Chronobiology, Woolcock Institute of Medical Research, Macquarie University, Sydney, NSW, Australia; NHMRC Centre of Research Excellence to Optimise Sleep in Brain Ageing and Neurodegeneration (CogSleep CRE), Sydney, NSW, Australia; CIRUS Centre for Sleep and Chronobiology, Woolcock Institute of Medical Research, Macquarie University, Sydney, NSW, Australia; Faculty of Medicine and Health, University of Sydney, Sydney, NSW, Australia; Department of Respiratory and Sleep Medicine, Royal Prince Alfred Hospital, Camperdown, Sydney, NSW, Australia; CIRUS Centre for Sleep and Chronobiology, Woolcock Institute of Medical Research, Macquarie University, Sydney, NSW, Australia; NHMRC Centre of Research Excellence to Optimise Sleep in Brain Ageing and Neurodegeneration (CogSleep CRE), Sydney, NSW, Australia; Faculty of Medicine and Health, University of Sydney, Sydney, NSW, Australia; Department of Respiratory and Sleep Medicine, Royal Prince Alfred Hospital, Camperdown, Sydney, NSW, Australia; School of Psychology, Faculty of Science, Brain and Mind Centre and Charles Perkins Centre, University of Sydney, Sydney, NSW, Australia; NHMRC Centre of Research Excellence to Optimise Sleep in Brain Ageing and Neurodegeneration (CogSleep CRE), Sydney, NSW, Australia; School of Psychology, Faculty of Science, Brain and Mind Centre and Charles Perkins Centre, University of Sydney, Sydney, NSW, Australia; CIRUS Centre for Sleep and Chronobiology, Woolcock Institute of Medical Research, Macquarie University, Sydney, NSW, Australia; NHMRC Centre of Research Excellence to Optimise Sleep in Brain Ageing and Neurodegeneration (CogSleep CRE), Sydney, NSW, Australia; School of Psychological Sciences, Macquarie University, Sydney, NSW, Australia

**Keywords:** Sleep-disordered breathing, electroencephalography, EEG power spectra, learning, cognition

## Abstract

**Study Objectives:**

To compare overnight declarative memory consolidation and non-rapid eye movement (NREM) sleep electroencephalogram (EEG) oscillations in older adults with obstructive sleep apnea (OSA) to a control group and assess slow-wave activity (SWA) and sleep spindles as correlates of memory consolidation.

**Methods:**

Forty-six older adults (24 without OSA and 22 with OSA) completed a word-pair associate's declarative memory task before and after polysomnography. Recall and recognition were expressed as a percentage of the morning relative to evening scores. Power spectral analysis was performed on EEG recorded at frontal (F3-M2, F4-M1) and central (C3-M2, C4-M1) sites. We calculated NREM absolute slow oscillation (0.25–1 Hz) and delta (0.5–4.5 Hz) EEG power, and slow (11–13 Hz) spindle density (number of events per minute of N2 sleep) and fast (13–16 Hz) spindle density.

**Results:**

There were no significant differences in overnight recall and recognition between OSA (mean age 58.7 ± 7.1 years, apnea–hypopnea index (AHI) 41.9 ± 29.7 events/hour) and non-OSA (age 61.1 ± 10.3 years, AHI 6.6 ± 4.2 events/hour) groups. The OSA group had lower fast spindle density in the frontal region (*p* = 0.007). No between-group differences in SWA were observed. In the Control group, overnight recognition positively correlated with slow spindle density in frontal (rho = 0.555, *p* = 0.020) and central regions (rho = 0.490, *p* = 0.046). Overnight recall was not related to SWA or spindle measures in either group.

**Conclusions:**

Older adults with OSA had deficits in fast sleep spindles but showed preserved overnight declarative memory consolidation. It is possible that compensatory mechanisms are being recruited by OSA patients to preserve declarative memory consolidation despite the presence of sleep spindle deficits.

## Graphical abstract

**Graphical Abstract:**
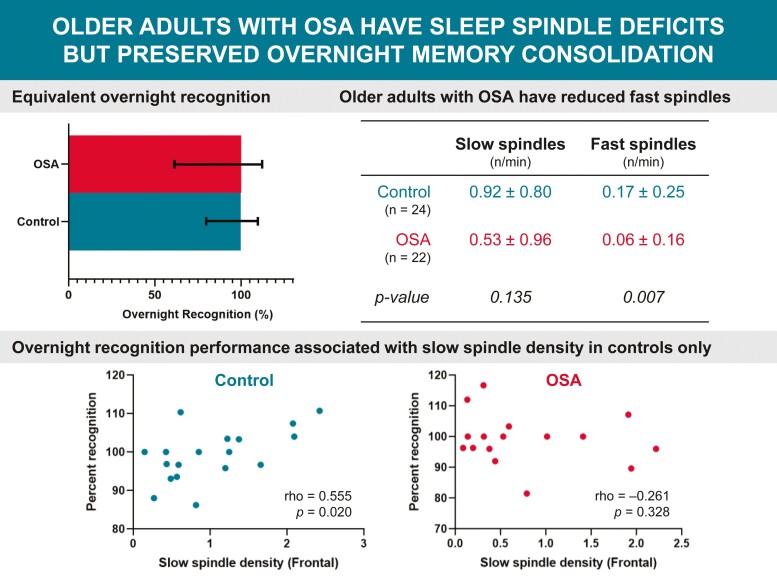

**Statement of Significance**

Prior studies have shown impaired overnight declarative memory consolidation in patients with untreated obstructive sleep apnea (OSA). Other studies have also reported deficits in sleep spindles and altered slow-wave activity. However, no prior studies have examined associations between overnight declarative memory and both sleep spindles and slow waves in older adults with OSA. Our findings suggest that overnight memory consolidation is intact in older adults with OSA despite deficits in sleep spindles. Fewer sleep spindles but intact memory consolidation in the OSA group suggests potential compensatory mechanisms may preserve favorable conditions for overnight consolidation of declarative memories.

## Introduction

Obstructive sleep apnea (OSA) is a common sleep breathing disorder affecting an estimated 23.4% of women and 49.7% of men with evidence suggesting an elevated prevalence with increasing age [1, 2]. Untreated OSA has adverse consequences on daytime functioning and cognition with impairments observed across several cognitive domains such as attention, processing speed, memory, visuospatial abilities, and executive functions [3, 4]. Using conventional neuropsychological assessment to explore the impact of OSA on different aspects of memory, synthesized findings from a meta-review demonstrated deficits in delayed verbal and visual long-term memory [3]. However, there are limited studies employing tasks to examine overnight memory consolidation in OSA, particularly in older adults. Given the high prevalence of OSA in older populations [2], and growing evidence suggesting OSA is associated with an increased risk for developing dementia [5, 6], it is crucial to understand the potential cognitive impact of untreated OSA in the older population.

OSA disrupts sleep architecture due to associated sleep fragmentation and intermittent hypoxia. Deficits in non-rapid eye movement (NREM) sleep electroencephalogram (EEG) oscillations including reduced sleep spindles [7, 8] and altered slow-wave activity (SWA) [9, 10] have been previously reported in OSA. These EEG oscillations are putatively involved in overnight memory consolidation processes [11–13], potentially serving as sensitive electrophysiological markers of memory impairment in OSA [14, 15]. In healthy young adults, sleep spindle density and slow frequency EEG activity (<1 Hz) are enhanced during sleep after intense associative learning of word pairs, as compared with a non-learning control condition [16, 17].

Relative to the body of work examining overnight memory consolidation in young healthy adults, there is limited work examining this process in OSA. Four OSA case–controlled studies assessed procedural memory using a motor sequence task [18, 19], mirror tracing task [20], and an alternating serial reaction time task [21]. They found that overnight consolidation was impaired in both young [18] and middle-aged patients with moderate OSA [19–21], relative to controls. These findings suggest that overnight procedural memory consolidation is impaired in people with untreated OSA.

Of the case–controlled studies that have assessed overnight declarative memory in OSA [20, 22–25], two studies in children aged 16 years and younger [22, 25] found that children with OSA performed poorer on overnight recall and studies in adults with OSA reported deficits in verbal memory [20, 23, 24], but not visuospatial memory [20].

Of the studies that have examined the relationship between sleep architecture and overnight declarative memory consolidation in untreated OSA, poorer overnight consolidation was related to deficits in N2 sleep spindles (12–15 Hz) in children [25] and reduced time spent in slow-wave sleep (SWS) in adults [23, 24]. However, there is a paucity of research exploring overnight declarative memory consolidation and its relationship with NREM sleep microarchitecture in older adults with OSA. To the best of our knowledge, no prior studies have explored declarative memory consolidation in relation to slow-wave activity and slow and fast sleep spindles in adults with OSA, despite the demonstrated spindle deficits in this population. The two types of sleep spindles appear to have topographical and functional differences with slow spindles (11–13 Hz) primarily observed in the frontal brain region, and fast spindles (13–16 Hz) prominent in the central and parietal regions [26]. In healthy young adults, slow sleep spindles were associated with overnight declarative memory performance [27] and fast sleep spindles positively correlated with procedural memory [28].

Therefore, this study aims to examine overnight declarative memory consolidation and sleep spindles and SWA in older people with and without OSA and examine associations between these NREM sleep EEG oscillations and memory. We hypothesized that patients with OSA would have poorer memory consolidation, reduced sleep spindles, and SWA (slow oscillation [SO] and delta power) relative to controls, and that worse overnight memory performance would correlate with greater NREM sleep EEG deficits.

## Methods

### Participants

OSA participants over the age of 50 years were recruited from the Woolcock Sleep Clinic, Woolcock Institute of Medical Research, Sydney, NSW, Australia. OSA was defined by an apnea–hypopnea index (AHI) of ≥15/hours diagnosed by attended in-laboratory polysomnography. OSA participants were newly diagnosed and were not receiving OSA treatment while participating in the study. Controls aged over 50 years were recruited from the Healthy Brain Ageing Clinic, Sydney or in response to a community advertisement.

Participants were excluded if they had major depression, bipolar disorder, schizophrenia, neurological disorder (e.g. Parkinson’s disease), previous head injury with loss of consciousness for more than 30 minutes, dementia or a mini-mental state examination (MMSE) score less than 24, transmeridian travel within the past week, alcohol or illicit drug misuse or were currently/ regularly taking sedatives, and hypnotics and other sleep-affecting medications. All procedures were approved by University of Sydney Human Research Ethics Committee (protocol number: 2016_410) and were carried out at the Woolcock Institute of Medical Research. All participants provided written informed consent in accordance with ethical guidelines.

### Procedure

Participants arrived at the sleep laboratory in the early evening (between 06:00 pm and 07:00 pm) for an overnight polysomnography. Lights out time was scheduled at 10: 00 pm and lights on time was at 06:00 am.

Participants completed the Pittsburgh Sleep Quality Index, Epworth sleepiness scale, and insomnia severity index questionnaires and the MMSE. The Karolinska sleepiness scale was administered prior to lights out and after lights on.

The learning phase of the memory task occurred in the evening 1–2 hours before lights out. Morning memory testing occurred 1 hour after lights were on.

### Word-pair associates task

Declarative verbal memory was measured using a 32-item word-pair task which incorporated 16 semantically related (e.g. actor–movie) word pairs that would facilitate pre-sleep encoding as well as 16 semantically unrelated (e.g. salt–call) word pairs [29, 30].

The words comprising the pairs ranged from three to eight letters and one to three syllables in length. Two lists were prepared, which made up set 1 and set 2 of the task, respectively. Half of the participants completed each set to control for any retention effects related to the pairs themselves. In both sets, for the semantically related pairs, half of the 32 words were paired with their second most frequently produced target. The most frequently produced target served as a foil (distractor) in the multiple-choice testing (recognition) phase of the task. The other half of the 32 words were paired semantically unrelated words, also taken from Nelson et al. (2004) [29] and paired together using a random number generator but checked to ensure no semantic association between pairs. The targets most frequently produced in response to all first words of the pairs served as foils in the recognition phase.

#### Encoding phase.

Participants were randomly presented with each word-pair on a computer screen. During each learning trial, a word-pair was displayed for 5 seconds and following the presentation of the word-pair, participants were asked to count and say out loud how many syllables in each of the word-pair for one-half of the word-pairs then put the two words into a sentence for the other half of the word-pairs. This was aimed to manipulate the depth of processing with syllables representing a shallower level of processing and formation of sentences representing a deeper level of processing.

#### Evening testing phase.

This consisted of two parts: cued recall and recognition (multiple-choice test). Immediate recall was tested after all 32-word pairs were presented whereby participants were shown the first word of the pair and asked to say aloud the second word. Participants were given 12 seconds to respond. For the recognition multiple-choice test component, the first word of each pair was presented on the computer screen alongside four options for the corresponding word: the correct word, a semantically related but incorrect word, an unrelated word, and a word containing the same number of syllables as the correct pair. Participants were asked to say the correct word and were given 8 seconds to respond. Following the response, participants were shown the correct pair for 3 seconds. Both encoding and evening testing phases were repeated until a criterion of 60% (19 pairs) was met or participants completed both phases four times.

#### Morning testing phase.

Both recall and recognition tests were performed once in the morning following the overnight sleep opportunity and after being awake for one hour.

#### Memory outcome measures.

The evening pre-sleep scores for recall and recognition were calculated as the total number of correct answers in the last recall and recognition test. The morning post-sleep score was calculated as the total number of correct answers in the recall and recognition test. The memory consolidation outcomes were *percent overnight recall* (morning recall score divided by evening recall score multiplied by 100) and *percent overnight recognition* (morning recognition score divided by evening recognition score multiplied by 100).

### Sleep recording

Polysomnography data were collected using a standardized montage (six-channel electroencephalogram recorded referentially at scalp positions: F3-M2, F4-M1, C3-M2, C4-M1, O1-M2, and O2-M1); electrooculogram (left and right outer canthi); and chin electromyogram, electrocardiogram , nasal airflow pressure (nasal cannula), thoracic and abdominal respiratory effort, finger pulse oximetry, body position, and leg electromyogram. EEG data were sampled at 200 Hz (Alice 5) or 512 Hz (Embla Titanium) and a 50 Hz notch filter was applied to reduce mains interference. EEG and electrooculogram electrodes were referenced to the contralateral mastoid electrode and impedances were below 5 kΩ at the start of the recording. Sleep stages and respiratory events were manually scored by trained sleep technicians according to standardized AASM 2.2 criteria [31].

### EEG artifact processing

All polysomnographic recordings were exported into standardized digital European Data Format prior to artifact detection and all subsequent quantitative EEG analyses. All night polysomnography recordings were subjected to automated EEG artifact processing. An algorithm identified artefactual EEG data at a resolution of 5-second epochs based on previously validated artifact detection threshold parameters [32]. The automatic algorithm developed was based on a statistical distribution of standard deviation (SD)s of EEG amplitudes in each 5-second epoch where two optimal artifact detection threshold parameter values (λ1 for S1, S2, REM and WASO, and λ2 for SWS) were systematically determined and subsequently tested with a high level of accuracy when compared to a reference standard manual artifact detection method [32]. Contaminated 5-second epochs were subsequently excluded from EEG analysis.

### EEG spectral analysis

Artifact-free epochs were analyzed for power spectra using a standard fast Fourier transform with a rectangular weighted window [33] for each non-overlapping 5-second epoch of EEG for each individual frontal (F3-M2 and F4-M1) and central (C3-M2 and C4-M1) channel, using a technique previously described [32]. This routine requires the total number of data points in each epoch to be in the form of a power of two, i.e. 512 (2^9^) data points. For EEG data sampled at a rate of 200 Hz, i.e. 1000 data points for a 5-second epoch, the fast Fourier transform was performed twice, to 512 data points selected from the beginning and end of each epoch. The resulting mean value for the epoch gives a greater weight to the middle data points.

We calculated absolute spectral power (*µ*V^2^) by summing the binned values within the frequency ranges of 0.25 to 1 Hz inclusive, and 0.5 and 4.5 Hz inclusive for the SOs and delta bands, respectively for each 5-second epoch. The absolute EEG power for each sleep-staged 30-second epoch of the polysomnography recording was calculated by averaging data from up to six artifact-free 5-second epochs of EEG that comprised that 30-second recording segment. The weighted-average spectra power within the defined frequency bands was then computed for NREM (N2 and N3) sleep stages by averaging the absolute spectral power for all 30-second epochs for the given sleep stage.

### Automated sleep spindle detection algorithm

The automated sleep spindle detection tool was developed and written in Java, version 1.6 (Oracle, Santa Clara, CA, USA). First, a 128-order band-passing Finite-Impulse-Response filter (11–16 Hz) is applied to the raw EEG signal. Then, a Hilbert transformation was applied to extract envelopes (duration) of spindles based on amplitude thresholds. The resulting output included sleep spindle density (expressed as the number of spindles per min of N2 sleep) which comprises slow (11–13 Hz, events p/min) and fast (13–16 Hz, events p/min) spindle densities. The automated spindle detection algorithm has been validated in sleep apnea and other clinical populations [30, 34].

### Statistical analysis

All statistical analyses were conducted using Statistical Packages for Social Sciences (SPSS v25.0.0; IBM Corp, 2020). A *P*-value of < 0.05 was considered significant. Outcome and demographic variables were inspected visually to check for normality and potential outliers (points that extend more than 3 times the interquartile range from the edge of the box plots). Log transformations were performed where applicable. Independent samples *t*-test was used for between-group comparisons of demographic, memory, PSG, and sleep EEG measures when variances between the two groups were equal, Mann–Whitney U test was used for unequal variances, and the Chi-square test for categorical variables. Effect sizes and 95% confidence limits were also calculated to provide a common standardized scale to quantify the difference in declarative memory between groups. The formula for the effect size was calculated as the difference between the group means (mean of OSA group – mean of Control group) divided by the square root of the pooled SD. As there were unequal group sizes, the pooled SD was calculated as follows: (SD_control_^2^ × (*n*_control_ − 1)) + (SD_OSA_^2^ × (n_OSA_−1))/ (*n*_control_ + n_OSA_ − 2).

A mixed model analysis of variances (ANOVA) with the repeated measurement factor test session (evening and morning), between-subject factor group (OSA patients and controls) was used to test for differences in memory performance. To assess the relationships between sleep EEG measures (SO and delta EEG power, and sleep spindle densities) and the memory outcomes (memory consolidation: percent overnight recall and percent overnight recognition) Pearson’s correlation coefficient (r) was used for normally distributed variables and Spearman’s rank order correlation (rho) for non-normally distributed variables. Correlations with a *P-*value < 0.05 were further examined using partial correlation to control for the confounder of age. No adjustments were made for multiple comparisons. Quantitative EEG measures computed for the individual channels of C3-M2 and C4-M2 were averaged to derive central brain region measures of SO power, delta power, slow spindle density and fast spindle density, and EEG measures from F3-M2 and F4-M1 were averaged to derive frontal brain region measures.

## Results

### Participant demographics

Forty-six older adults over the age of 50 years were enrolled in this study. Demographic data for the OSA and Control groups are shown in Table 1. Groups were not significantly different in age, gender, body mass index, MMSE, Pittsburgh Sleep Quality Index, and subjective sleepiness ratings (Karolinska sleepiness scale) in the evening and morning. However, the OSA group had significantly higher subjective sleepiness (Epworth sleepiness scale) and greater insomnia symptoms (insomnia severity index).

**Table 1.** Participant Demographics

	Control	OSA	Test statistic	*P-Value*
*n* (M/F)^b^	24 (11/13)	22 (8/14)	0.3	0.235
Age (years)	61.1 ± 10.3 (53–79)	58.7 ± 7.1 (58–73)	0.7	0.442
Body mass index (kg/m^2^)^a,c^	25.2 ± 12.6 (18.7–46.2)	27.5 ± 8.5 (23.1–34.2)	34	0.505
Mini-mental state examination^a,c^	30.0 ± 1.0 (26–30)	30.0 ± 0.0 (29–30)	188	0.077
Pittsburgh sleep quality index	5.0 ± 2.9 (2–10)	5.5 ± 2.4 (3–12)	0.7	0.480
Epworth sleepiness scale	5.7 ± 3.1 (2–12)	9.0 ± 3.9 (2–17)	−3.1	0.003*
Insomnia severity index	6.8 ± 4.7 (1–12)	9.8 ± 5.1 (2–19)	−2.0	0.047*
Karolinska sleepiness scale, evening	3.7 ± 2.1 (1–7)	3.2 ± 1.9 (1–7)	0.8	0.376
Karolinska sleepiness scale, morning	4.6 ± 1.7 (2–7)	4.3 ± 1.3 (3–5)	0.6	0.524

Data presented as mean±SD (min–max). * *p* < 0.05. Independent samples *t*-test was used for all variables unless stated otherwise. ^a^Mann–Whitney U test, ^b^Chi-square goodness of fit test was conducted, ^c^Median (interquartile range) presented due to non-normally distributed data.

### Word-pair task

Two participants from each group were excluded from the analysis of the word-pair task during the recall phase as they failed to reach a criterion of 60% correct and did not complete 4 trials in the evening and one further participant from the control group was excluded from the recognition phase as that participant declined to be assessed. As such the total sample analyzed for the primary outcome of percent overnight recall was 22 Controls and 20 OSA patients, and the total numbers assessed for percent overnight recognition was 21 Controls and 20 OSA patients.

**Figure 1.**
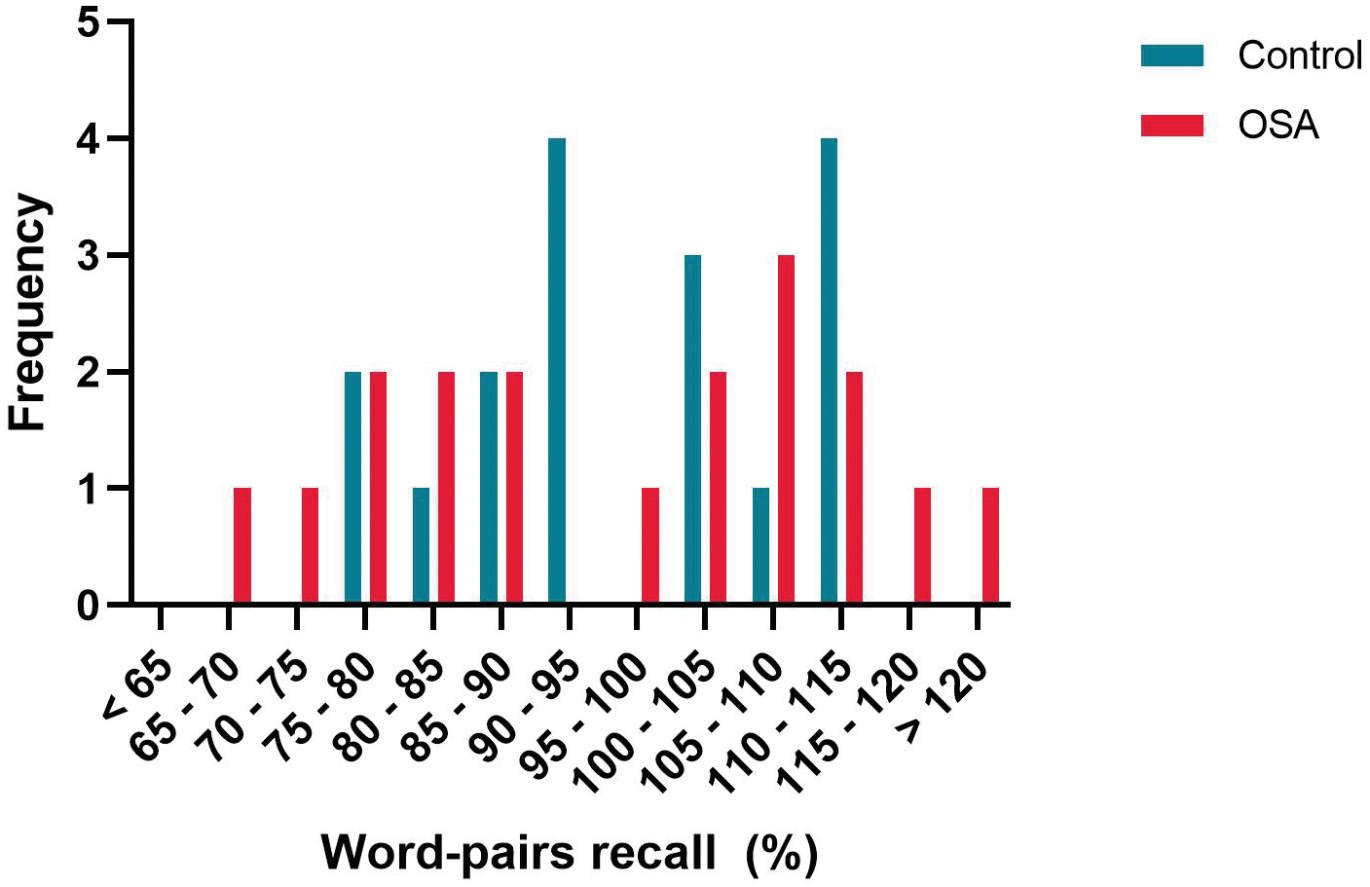
Frequency distribution of percentage of word-pairs recalled for both OSA and Control groups. There were no differences between groups. Some participants from both groups demonstrated enhancement of recall performance post-sleep (greater than 100% recall).

Percent overnight recall and percent overnight recognition did not differ between Controls and OSA groups: *t* (40) = −0.012, *p* = 0.990 vs. *t* (39) = −0.090, *p* = 0.93, Table 2 and Figure 1. There were no between-group differences in the number of word pairs learned in the evening (evening score) or in the number of word pairs recalled or recognized in the morning. For morning recall, the Control group on average recalled 20.9 words (SD = 4.7) which were not statistically different from the OSA group who on average recalled 20.8 words (SD = 4.5) (*p* = 0.94) (Figure 1). A mixed model ANOVA revealed no main effects for group (Controls vs. OSA: F = 0.000, *p* = 0.998), main effect for test session (evening vs. morning: F = 3.209, *p* = 0.081), or interaction between group × condition (F = 0.057, *p* = 0.813). The effect sizes for the memory measures are shown in Table 2.

**Table 2.** Between-Group Comparisons of Declarative Memory Performance (Word-Pair Task)

	Control	OSA	*df*	*Effect size (95% CIs)*	*P-Value*
Evening recall,/32	21.6 ± 2.8 (19–28)	21.7 ± 2.7 (19–27)	40	0.04 (−0.59, 0.66)	0.90
Morning recall,/32	20.9 ± 4.7 (10–27)	20.8 ± 4.5 (14–30)	40	−0.02 (−0.65, 0.60)	0.94
Evening recognition,/32	28.6 ± 2.1 (24–32)	27.9 ± 2.6 (24–32)	40	−0.30 (−0.93, 0.33)	0.31
Morning recognition,/32	28.6 ± 2.7 (22–32)	27.9 ± 3.2 (22–32)	39	−0.24 (−0.87, 0.40)	0.41
Percent overnight recall	96.0 ± 14.6 (52.6–114.4)	96.0 ± 15.6 (66.7–129.4)	40	0.00 (−0.62, 0.62)	0.99
Percent overnight recognition	99.8 ± 6.4 (86.2–110.7)	100.0 ± 7.9 (68.0–116.7)	39	0.03 (−0.60, 0.66)	0.93
Overnight related recall (%)	100.5 ± 13.7 (69.2–127.3)	96.5 ± 10.0 (76.9–114.3)	40	−0.33 (−0.96, 0.29)	0.29
Unrelated recall (%)	90.4 ± 30.6 (25.0–137.5)	98.2 ± 35.0 (50.0–200.0)	40	0.24 (−0.39, 0.86)	0.45
Deep recall (%)	96.5 ± 12.3 (66.7–120.0)	96.1 ± 14.5 (62.5–123.1)	40	−0.03 (−0.65, 0.59)	0.94
Shallow recall (%)	99.8 ± 22.8 (40.0–150.0)	97.0 ± 19.5 (66.7–130.0)	40	−0.13 (−0.76, 0.49)	0.67

Data presented as mean±SD (min–max). df, degrees of freedom; CIs, confidence intervals. Independent samples *t*-test was used for all variables.

### Polysomnography

There were no significant between-group differences in total sleep time, WASO, sleep efficiency, or sleep stage distribution (see Table 3). As expected, the OSA group had worse OSA disease severity metrics with a higher AHI and arousal index, and lower minimum oxygen saturation levels.

**Table 3.** Overnight Polysomnography

	Control	OSA	*P-value*
Time in bed (min)	426.3 ± 31.6 (375.7–532.0)	435.5 ± 34.8 (394.5–634.0)	0.355
Sleep efficiency (%)	80.0 ± 11.6 (43.6–92.0)	76.5 ± 17.2 (40.8–97.4)	0.420
Total sleep time (min)	344.1 ± 54.5 (175.0–418.5)	337.2 ± 79.2 (187.5–451.0)	0.734
Wake after sleep onset (min)	70.2 ± 41.5 (23.0–219.5)	76.4 ± 56.6 (9.5–205.0)	0.673
N1 (min)	18.2 ± 18.2 (1.5–80.0)	27.5 ± 20.7 (6.0–92.50)	0.119
N2 (min)	168.0 ± 49.5 (60.0–261.0)	177.5 ± 57.0 (97.5–280.6)	0.557
N3 (min)	87.2 ± 42.6 (1.5–180.5)	68.3 ± 30.1 (10.0–121.0)	0.095
NREM (min)	275.5 ± 40.3 (136.0–313.5)	274.8 ± 56.8 (143.0–332.5)	0.958
REM (min)	67.9 ± 27.0 (10.0–124.5)	62.9 ± 26.3 (22.5–103.5)	0.526
AHI (events/hour)	6.6 ± 4.2 (1.2–14.1)	41.9 ± 29.7 (15.0–128.4)	<0.001***
Minimum oxygen saturation (%)	89.2 ± 3.7 (81.0–95.0)	79.7 ± 12.8 (34.0–92.0)	0.001**
Total EEG arousal index (events/hour)	13.4 ± 5.2 (3.3–24.5)	39.4 ± 27.5 (9.4–120.1)	<0.001***

NREM, non-rapid eye movement; REM, rapid eye movement; AHI, apnea–hypopnea index. Data presented as mean±SD (min–max). ** *p* < 0.01, ***p < 0.001. Independent samples *t*-test was used for all variables.

### NREM sleep microarchitecture

Fast spindle density during N2 was lower in the OSA group in the frontal region (OSA vs. controls: 0.06 ± 0.16 vs. 0.17 ± 0.25, *p* = 0.007) but did not reach significance in the central region (0.19 ± 0.65 vs. 0.57 ± 1.01, *p* = 0.130). No significant between-group differences were observed in slow spindle density during N2 in the frontal region (OSA vs. control: 0.53 ± 0.96 vs. 0.92 ± 0.80, *p* = 0.135) or in the central region (0.28 ± 0.38 vs. 0.38 ± 0.52, *p* = 0.152). No between-group differences in SO or delta EEG power during NREM sleep were observed (see Table 4).

**Table 4.** Between-Group Comparisons of Absolute EEG Power in the SO (0.25–1.0 Hz) and Delta (0.5–4.5 Hz) Frequency Ranges During NREM Sleep, and Fast and Slow Spindle Densities During N2 Sleep

	EEG sleep measures	Control	OSA	*P-value*
Frontal	Slow oscillation EEG power in NREM (*n* = 39)	187.05 ± 132.91 (36.05–378.04)	216.11 ± 236.14 (31.59–1049.09)	0.920
	Delta EEG power in NREM (*n* = 39)	284.96 ± 206.06 (46.39–929.95)	308.39 ± 166.08 (93.87–520.06)	0.800
	Slow spindle density in N2^a,b^ (*n* = 39)	0.92 ± 0.80 (0.27–2.09)	0.53 ± 0.96 (0.09–2.22)	0.135
	Fast spindle density in N2^a,b^ (*n* = 39)	0.17 ± 0.25 (0.03–2.76)	0.06 ± 0.16 (0.00–0.67)	0.007**
Central	Slow oscillation EEG power in NREM (*n* = 41)	183.41 ± 124.73 (37.58–330.67)	176.48 ± 206.18 (30.94–975.42)	0.679
	Delta EEG power in NREM (*n* = 41)	246.98 ± 136.42 (68.19–634.84)	283.87 ± 171.14 (87.71–732.52)	0.667
	Slow spindle density in N2^a,b^ (*n* = 40)	0.38 ± 0.52 (0.02–1.57)	0.28 ± 0.38 (0.06–0.99)	0.152
	Fast spindle density in N2^a,b^ (*n* = 40)	0.57 ± 1.01 (0.14–3.54)	0.19 ± 0.65 (0.03–1.29)	0.130

*p < 0.05, **p < 0.01. Data presented mean±SD unless stated otherwise. Independent samples *t*-test was used unless stated otherwise. ^a^Median (interquartile range) presented due to non-normally distributed variables. ^b^Mann–Whitney U test was used.

### Associations between sleep microarchitecture measures and overnight memory consolidation

**Figure 2.**
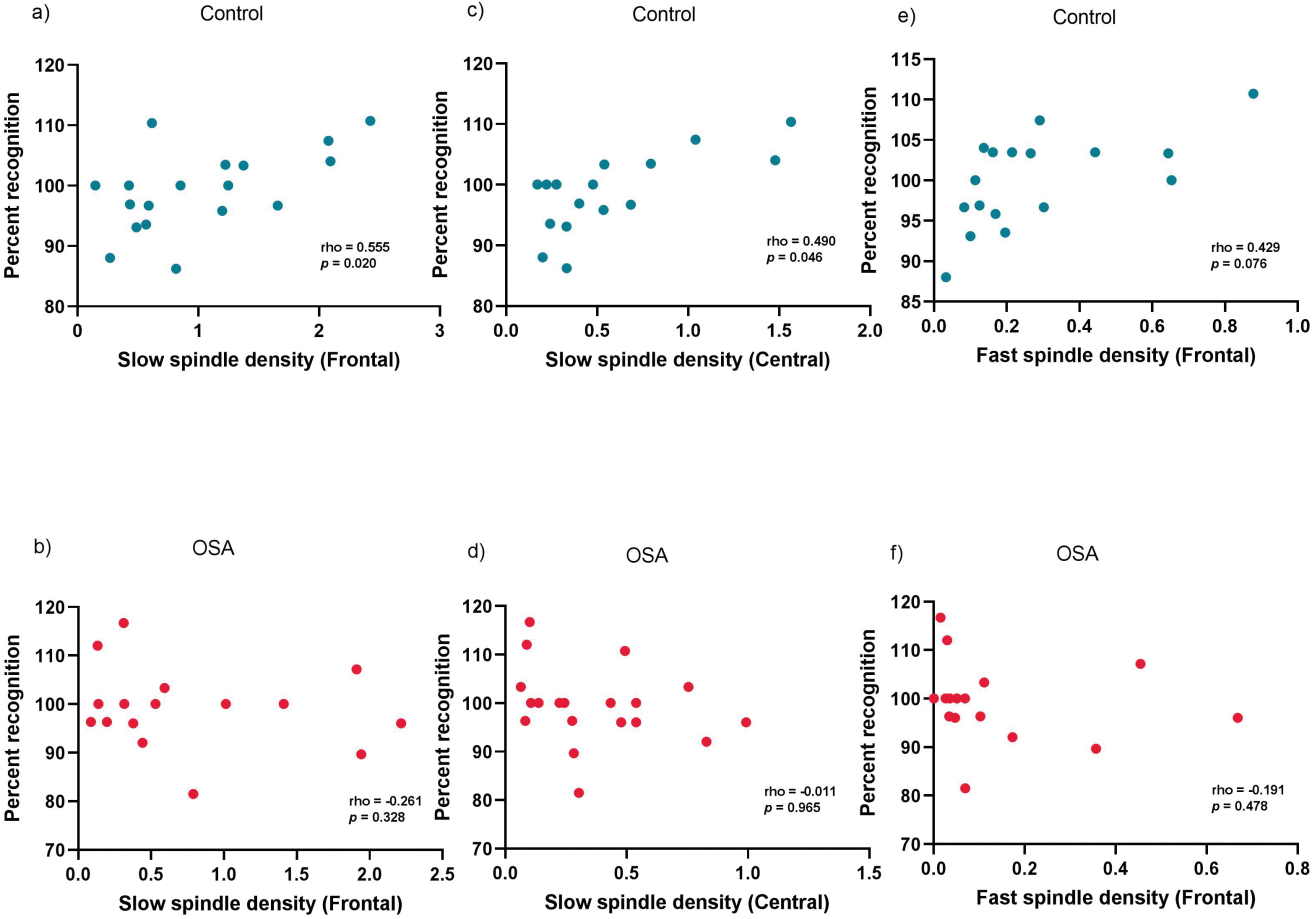
Scatterplots of associations using Spearman’s rho between percent overnight recognition and frontal slow spindle density (panels a and b); central slow spindle density (panels c and d); and frontal fast spindle density (panels e and f) in control and OSA groups. Spearman’s rho was used for all correlations.

In the Control group, overnight percent recognition positively correlated with slow spindle density in the frontal (Spearman’s rho = 0.555, *p* = 0.020) and central regions (rho = 0.490, *p* = 0.046), Figure 2. There was also a moderate sized, but trend association observed with frontal fast spindle density (rho = 0.429, *p* = 0.076). The association between central slow spindle density and percent overnight recognition remained significant after adjusting for age (see Table 5). There were no significant associations between percent overnight recall and slow or fast spindle density. Overnight memory outcomes were not associated with SWA (NREM SOs and delta EEG power) in the control group.

**Table 5.** Associations Between NREM Microarchitecture Metrics and Overnight Percent Recall and Recognition on the Word-Pair Associates Task.

Overnight recall			
		Control	OSA
** Frontal Region**	Slow oscillation EEG power in NREM (*n* = 32)	*r* = 0.266	*r* = 0.261
		*p* = 0.271	*p* = 0.328
	Delta EEG power in NREM (*n* = 37)	*r* = −0.025	*r* = 0.098
		*p* = 0.918	*p* = 0.719
	Slow spindle density in N2 (*n* = 35)	rho = 0.281	rho = 0.232
		*p* = 0.243	*p* = 0.388
	Fast spindle density in N2 (*n* = 35)	rho = 0.177	rho = 0.171
		*p* = 0.469	*p* = 0.526
Central region	Slow oscillation EEG power in NREM (*n* = 37)	r = 0.269	*r* = −0.036
		*p* = 0.281	*p* = 0.884
	Delta EEG power in NREM (*n* = 37)	r = 0.048	r = 0.070
		*p* = 0.851	*p* = 0.777
	Slow spindle density in N2 (*n* = 37)	rho = 0.359	rho = 0.325
		*p* = 0.143	p = 0.175
	Fast spindle density in N2 (*n* = 37)	rho = −0.062	rho = 0.326
		*p* = 0.807	*p* = 0.173
Overnight recognition			
Frontal region	Slow oscillation EEG power in NREM (*n* = 36)	*r* = −0.189	*r* = 0.081
		*p* = 0.452	*p* = 0.766
	Delta EEG power in NREM (*n* = 36)	*r* = 0.125	*r* = −0.138
		*p* = 0.622	*p* = 0.610
	Slow spindle density in N2 (*n* = 34)	rho = 0.555	rho = −0.261
		*p* = 0.020^*^	*p* = 0.328
		partial rho = 0.432	
		*p* = 0.108	
	Fast spindle density in N2 (*n* = 34)	rho = 0.429	rho = −0.191
		*p* = 0.076	*p* = 0.478
Central region	Slow oscillation EEG power in NREM (*n* = 36)	*r* = −0.129	*r* = −0.012
		*p* = 0.623	*p* = 0.962
	Delta EEG power in NREM (*n* = 36)	*r* = 0.101	*r* = 0.035
		*p* = 0.970	*p* = 0.888
	Slow spindle density in N2^a^ (*n* = 36)	rho = 0.490	rho = −0.011
		*p* = 0.046*	*p* = 0.965
		partial rho = 0.728	
		*p* = 0.002**	
	Fast spindle density in N2 (*n* = 36)	rho = 0.280	rho = −0.070
		*p* = 0.276	*p* = 0.777

*p < 0.05, **p < 0.01. Spearman’s correlation coefficient (rho) was used for spindle densities due to non-normal distribution and Pearson’s correlation coefficient (r) for normally distributed SO EEG power and delta EEG power after log transformation. ^a^Associations remained significant after accounting for age using partial correlation. SO, frequency range 0.25–1 Hz; delta, 0.5–4.5 Hz.

There were no significant associations between any sleep microarchitecture measures of interest and overnight memory outcomes within the OSA group.

### Exploratory analysis

Post hoc analysis was conducted within groups to explore correlations between the duration spent in both SWS (mins and %) and overnight memory outcomes. Neither percent overnight recall nor percent overnight recognition were associated with SWS in the Control or OSA groups.

We conducted a post hoc sensitivity analysis on memory measures by changing the demarcation of OSA cases and controls using the criterion of AHI ≥ 10; however, the results did not change from those using the predetermined cut point of AHI ≥ 15 with no between-groups differences in memory performance observed.

## Discussion

This study assessed overnight declarative verbal memory consolidation and key aspects of NREM sleep microarchitecture putatively involved in memory consolidation in older people with and without OSA and examined associations between memory and NREM EEG oscillations. Contrary to our hypothesis, overnight memory consolidation assessed using a verbal word-pair task was not different between the OSA and age-matched control groups. However, the OSA group had deficits in sleep spindles with lower N2 fast spindle density in the frontal region. No between-group differences in NREM SO or delta EEG power were observed. Higher slow spindle density in central and frontal regions was associated with better overnight recognition in the control group, but not in the OSA group. Overnight recall was not significantly associated with sleep spindle or slow-wave EEG measures within either group.

In contrast to the studies investigating non-declarative memory in OSA [18, 19] which show deficits in procedural memory, our study shows that in moderate to severe OSA patients, overnight consolidation of declarative verbal memory appears to be intact. Our findings in older adults with OSA differ from the decrements observed in overnight declarative memory in a smaller study of 15 middle-aged (average age 46 years) patients with moderate OSA [20]. This discrepant finding may be explained by the different memory tasks used where our study adopted a 32-item verbal paired associate task compared to a visual and verbal memory task where participants were required to memorize a line-drawn path on a map [20]. Others that employed similar word-pair tasks have also shown OSA patients to perform more poorly than controls on overnight recall [23, 24]. However, in those studies, the OSA group spent a significantly shorter amount of time in N3 slow-wave sleep than the control group, a difference not evident in our OSA group. Moreover, morning retention on the verbal paired associate task correlated with the amount of slow-wave sleep in the prior work, possibly accounting for the discordant findings between studies. Another possible explanation for the lack of significant differences in memory performance between groups in the current study is the small sample size which was only powered to reliably detect a large effect size. It is also possible that the preserved overnight declarative memory performance observed in older adults with OSA in our study may be due to preconditioning and tolerance in the brain in response to repeated exposure to intermittent hypoxic insults [35]. Furthermore, brain adaptive responses in untreated OSA including hypertrophy of the hippocampus suggesting hippocampal neurogenesis [36], may compensate for brain injury in other regions and act to preserve cognitive function. Future studies investigating overnight memory consolidation in carefully matched older adults with and without OSA should employ neuroimaging to further elucidate the brain’s compensatory responses.

We did not observe a difference in SWA (NREM SO or delta EEG power) between OSA and control groups. Deficits in SWA in OSA patients have previously been observed [36] and greater SWA has been related to improved spatial memory in OSA [14], and declarative memory and procedural learning in healthy adults and people with schizophrenia [37]. Concordant with our findings, other OSA case–controlled studies did not show any significant differences in SWA during NREM between groups [7–9, 14]. Similar to previous work [25, 38], we observed deficits in sleep spindles in the OSA group. These findings suggest that loss of sleep spindles during N2 may be a trait NREM EEG marker in OSA patients [39].

Slow sleep spindle density during N2 was positively associated with overnight recognition in healthy older adults, similar to previous research investigating the relationship between overnight retention of declarative word pairs and slow sleep spindles in younger adults [11, 16] and children [25]. Despite the similarity of the memory task used in our study as compared to previous studies, our results demonstrate the complexity and importance of a differentiation between overnight recognition and recall in declarative verbal associate memory and their relation to sleep spindles. It has been unclear in the literature whether sleep spindles represent individual traits associated with learning and memory functions or whether they play a causal role in memory consolidation. Supporting trait-like features of sleep spindles, Hoedlmoser et al. [40] reported that N2 slow spindle activity was associated with general baseline cognitive abilities and learning capabilities on a declarative memory task but not overnight declarative memory consolidation. Our study cannot exclude the possibility N2 slow sleep spindles reflect baseline cognition because we did not measure intelligence quotients.

Contrary to our hypotheses, we did not observe any association between sleep spindles and overnight memory consolidation in OSA. Although there are numerous studies that report associations between spindle activity and declarative memory in healthy sleepers, this relationship in OSA patients is less clear. It is possible that sleep spindle density is a poor biomarker of declarative memory consolidation in this population and other morphological characteristics such as spindle event duration or frequency variation may be more sensitive measures [41]. The issue of whether sleep spindle subtypes are differentially associated with memory consolidation in OSA also remains unclear. Slower frequency spindles have been observed in OSA [7, 42] and though our slow spindle events were detected within the 11 to 13 Hz frequency range it is possible that a lower bound for this range may more reliably capture individual spindle events and measures of spindle densities in this population. Given the different spatial and temporal distributions of fast and slow spindles, it is possible that they play different roles in sleep-related memory processes. Increased slow sleep spindles correlated with improved verbal declarative memory performance in healthy young adults [11, 27]. A study on healthy children failed to replicate those findings. For instance, Hoedlmoser et al. [40] found a relationship between slow sleep spindle activity and general cognitive abilities (intelligence scores) and initial acquisition rate during encoding sessions (learning efficiency). However slow sleep spindle activity in this study was not associated with overnight improvement in declarative memory performance. The utility of metrics that quantify the multidimensional aspects of sleep spindles (e.g. slow vs. fast frequency spindles and spindle amplitude and duration), their topography and coupling with SOs in relation to declarative memory consolidation in OSA is unclear and future studies employing high-density EEG and advanced signal processing techniques are required to address this gap.

There are several limitations to this study. The absence of impaired declarative memory in the current sample may result from the selection criteria of the study which excluded significant comorbidities as secondary causes of cognitive deterioration, and the presence of mild OSA in the control group. However, there is no clear evidence that supports an association between mild OSA and neurocognitive function [43]. Furthermore, the number in each group was relatively small for our sample size despite being comparable to other published sleep EEG studies in patients with sleep disorders and as such, we did not adjust our statistical analysis for multiple comparisons. We did not measure sleep spindle activity during a baseline night and are unable to evaluate whether those with greater sleep spindle activity at baseline had better memory consolidation, and therefore could not adjust for any potential differences between groups. It is possible that compensatory mechanisms that may have been recruited by OSA patients to explain the preservation of declarative memory consolidation, a phenomenon that has been observed when assessing performance in other cognitive domains [44]. Recent studies have demonstrated that memory consolidation during sleep relies on the precisely timed interaction between SOs and sleep spindles [45, 46]. Specifically, the tight precision of fast sleep spindles and SOs coupling is key to enhance memory consolidation. We did not examine spindle and slow-wave coupling in our study [47], and future studies that investigate the distinct temporal coordination of slow waves and spindles and its correlation with overnight declarative memory consolidation in OSA are required. In addition, it is noteworthy that the daytime neuropsychological cognitive profile of the current sample is unknown. In patients with OSA, as assessed in the current sample, it is possible that poor memory consolidation in OSA could co-occur with more general neuropsychological dysfunction as observed in a study by Beaudin et al. [48], where they reported the presence of mild cognitive impairment in 55.3% of patients with moderate and severe OSA. This notion was further supported in another study showing that participants who exhibited daytime neuropsychological impairment (i.e. mild cognitive impairment) had poorer overnight memory consolidation [30]. The presence of other confounders such as duration of disease, cognitive reserve, and continuous positive airway pressure use may act as a mediator or protection against cognitive decline.

## Conclusion

Despite the sleep fragmentation and hypoxemia associated with sleep-disordered breathing, overnight declarative memory consolidation appeared to be preserved in this small group of older adults with untreated OSA. However, larger studies that are adequately powered are required to confirm this. Moreover, we observed deficits in sleep spindle activity but not SWA in the OSA group. In healthy older adults, we provide further evidence that indicates sleep spindles play a role in the consolidation of declarative memories, highlighting the potential of targeted interventions to boost spindles to enhance cognitive outcomes in older adults without OSA. Future studies should examine the temporal, spatial, and dynamic characteristics of sleep spindles and slow waves to better understand sleep-dependent declarative memory processes and possible compensatory mechanisms in OSA.

## Data Availability

The data underlying this article will be shared on reasonable request to the corresponding author.

## References

[1] Heinzer R , Va S, Marques-Vidal P, Marti-Soler H, et al. Prevalence of sleep-disordered breathing in the general population: the hypnolaus study. Lancet Respir Med. 2015;3(4):310–318. doi: 10.1016/S2213-2600(15)00043-0 25682233 PMC4404207

[2] Peppard PE , Young T, Barne JH, et al. Increased prevalence of sleep-disordered breathing in adults. Am J Epidemiol. 2013;177(9):1006–1014. doi: 10.1093/aje/kwa342 23589584 PMC3639722

[3] Bucks RS , Olaithe M, Eastwood P. Neurocognitive function in obstructive sleep apnoea: a meta-review. Respirology. 2013;18(1):61–70. doi: 10.1111/j.1440-1843.2012.02255.x 22913604

[4] Cross N , Lampit A, Pye J, Grunstein RR, Marshall N, Naismith SL. Is obstructive sleep apnoea related to neuropsychological function in healthy older adults? A systematic review and meta-analysis. Neuropsychol Rev. 2017;27(4):389–402. doi: 10.1007/s11065-017-9344-6 28484904

[5] Yaffe K , Laffan AM, Harrison SL, et al. Sleep-disordered breathing, hypoxia, and risk of mild cognitive impairment and dementia in older women. JAMA. 2011;306:613–619. doi: 10.1001/jama.2011.1115 21828324 PMC3600944

[6] Shi L , Chen S, Ma M, et al. Sleep disturbances increase the risk of dementia: a systematic review and meta-analysis. Sleep Med Rev. 2018;40:4–16. doi: 10.1016/j.smrv.2017.06.010 28890168

[7] Himanen SL , Virkkala J, Huupponen E, Hasan J. Spindle frequency remains slow in sleep apnea patients throughout the night. Sleep Med. 2003;4(3):229–234. doi: 10.1016/s1389-9457(02)00239-3 14592327

[8] Ondze B , Espa F, Dauvilliers Y, Billard M, Besset A. Sleep architecture, slow wave activity and sleep spindles in mild sleep disordered breathing. Clin Neurophysiol. 2003;114(5):8670874. 10.1016/s1388-2457(02)00389-9 12738432

[9] Heinzer G , Decary A, Sforza E, Petit D, Morisson F, Montplaisir J. Slow-wave activity in sleep apnea patients before and after continuous positive airway pressure treatment: contribution to daytime sleepiness. Chest. 2001;119(6):1807–1813. 11399708 10.1378/chest.119.6.1807

[10] Jones SG , Riedner, BA, Smith, RF. et al. Regional reductions in sleep electroencephalography power in obstructive sleep apnea: a high-density EEG study. Sleep. 2014;37(2):399–407. doi: 10.5665/sleep.3424 24497668 PMC3900624

[11] Lustenberger C , Wehrle F, Tüshaus L, Achermann P, Huber R. The multidimensional aspects of sleep spindles and their relationship to word-pair memory consolidation. Sleep. 2015;38(7):1093–1103. doi: 10.5665/sleep.4820 25845686 PMC4481015

[12] Marshall L , Born J. The contribution of sleep to hippocampus-dependent memory consolidation. Trends Cogn Sci. 2007;11:442–450. doi: 10.1016/j.tics.2007.09.001 17905642

[13] Walker MP. The role of slow wave sleep in memory processing. J Clin Sleep Med. 2009;5(2 suppl):S20–S26. 19998871 PMC2824214

[14] Mullins AE , Williams MK, Kam K, et al. Effects of obstructive sleep apnea on human spatial navigational memory processing in cognitively normal older adults. J Clin Sleep Med. 2021;17(5):939–948. doi: 10.5664/jcsm.9080 33399067 PMC8320476

[15] Stevens D , Leong CWY, Cheung H, et al. Sleep spindle activity correlates with implicit statistical learning consolidation in untreated obstructive sleep apnea patients. Sleep Med. 2021;86:126–134. doi: 10.1016/j.sleep.2021.01.035 33707093

[16] Gais S , Molle M, Helms K, Born J. Learning-dependent increases in sleep spindle density. J Neurosci. 2002;22:68306840–68306834. doi: 10.1523/jneurosci.22-15-06830.2002 PMC6758170 12151563

[17] Molle M , Marshall L, Gais S, Born J, Raichle M. Learning increases human electroencephalographic coherence during subsequent slow sleep oscillations. Proc Natl Acad Sci USA. 2004;101(38):13963–13968. 15356341 10.1073/pnas.0402820101 PMC518860

[18] Djonlagic I , Saboisky J, Carusona A, Stickgold R, Malhotra A. Increased sleep fragmentation leads to impaired off-line consolidation of motor memories in humans. PLoS One. 2012;7(3):e34106. doi: 10.1371/journal.pone.0034106 22470524 PMC3314699

[19] Landry S , Anderson C, Andrewartha P, Sasse A, Conduit R. The impact of obstructive sleep apnea on motor skill acquisition and consolidation. Sleep Med. 2014;10(5):491–496. doi: 10.5664/jcsm.3692 PMC4046361 24910549

[20] Kloepfer C , Riemann D, Nofzinger EA, et al. Memory before and after sleep in patients with moderate obstructive sleep apnea. J Clin Sleep Med. 2009;5(6):540–548. 20465021 PMC2792970

[21] Csabi E , Varszegi-Schulz M, Janacsek K, Malecek N, Nemeth D. The consolidation of implicit sequence memory in obstructive sleep apnea. PLoS One. 2014;9(10):e109010109010. doi: 10.1371/journal.pone.0109010 PMC4198077 25329462

[22] Kheirandish-Gozal L , De Jong MR, Spruyt K, Chamuleau SA, Gozal D. Obstructive sleep apnoea is associated with impaired pictorial memory task acquisition and retention in children. Eur Respir J. 2010;36(1):164–169. 20075057 10.1183/09031936.00114209

[23] Guo M , Igue M, Malhotra A, Stickgold R, Djonlagic I. The effect of obstructive sleep apnea on declarative memory consolidation. Sleep Med. 2013;14:e33–e34. doi: 10.1016/j.sleep.2013.11.041

[24] Djonlagic IE , Guo M, Igue M, Kishore D, Stickgold R, Malhotra A. Continuous positive airway pressure restores declarative memory deficit in obstructive sleep apnea. Am J Respir Crit Care Med. 2021;203(9):1188–1190. doi: 10.1164/rccm.202011-4253LE 33347378 PMC8314910

[25] Maski K , Steinhart E, Holbrook H, Katz ES, Kapur K, Stickgold R. Impaired memory consolidation in children with obstructive sleep disordered breathing. PLoS One. 2017;12(11):e0186915. doi: 10.1371/journal.pone.0186915 29095855 PMC5667754

[26] Fernandez LMJ , Luthi A. Sleep spindles: mechanisms and functions. Physiol Rev. 2020;100(2):805–868. doi: 10.1152/physrev.00042.2018 31804897

[27] Clemens Z , Fabó, D, Halász, P. Overnight verbal memory retention correlates with the number of sleep spindles. Neuroscience. 2015;132(2):529–535. doi: 10.1016/j.neuroscience.2005.01.011 15802203

[28] Tamaki M , Matsuoka T, Nittono H, Hori T. Fast sleep spindle (13-15 Hz) activity correlates with sleep-dependent improvement in visuomotor performance. Sleep. 2008;31(2):204–211. doi: 10.1093/sleep/31.2.204 18274267 PMC2225572

[29] Nelson DL , McEvoy CL, Schreiber TA. The university of south florida free association, rhyme, and word fragment norms. Behav Res Meth Instr. 2004;36(3):402–407. doi: 10.3758/BF03195588 15641430

[30] Lam A , Haroutonian C, Grummitt L, et al. Sleep-dependent memory in older people with and without MCI: the relevance of sleep microarchitecture, OSA, hippocampal subfields, and episodic memory. Cereb Cortex. 2021;31(6):2993–3005. doi: 10.1093/cercor/bhaa406 33565576

[31] Berry RB , Budhiraja R, Gottlieb DJ, et al. Rules for scoring respiratory events in sleep: update of the 2007 AASM manual for the scoring of sleep and associated events. deliberations of the sleep apnea definitions task force of the american academy of sleep medicine. J Clin Sleep Med. 2012;8(5):597–619. doi: 10.5664/jcsm.2172 23066376 PMC3459210

[32] D’Rozario AL , Dungan GC, 2nd, Banks, S. et al. 2015. An automated algorithm to identify and reject artefact for quantitative EEG analysis during sleep in patients with sleep-disordered breathing. Sleep Breath 19(2) 607–615. doi: 10.1007/s11325-014-1056-z 25225154

[33] Press W H. Numerical Recipes in FORTRAN: The Art of Computing. Cambridge, UK: Cambridge University Press; 1992.

[34] D’Rozario AL , Hoyos CM, Wong KKH, et al. Improvements in cognitive function and quantitative sleep EEG in OSA after six months of CPAP treatment. Sleep. 2022;45(6). doi: 10.1093/sleep/zsac013 PMC9189957 35029691

[35] Dirnagl U , Becker K, Meisel A. Preconditioning and tolerance against cerebral ischaemia: from experimental strategies to clinical use. Lancet Neurol. 2009;8(4):398–412. doi: 10.1016/S1474-4422(09)70054-7 19296922 PMC2668955

[36] Rosenzweig I , Kempton MJ, Crum WR, et al. Hippocampal hypertrophy and sleep apnea: a role for the ischemic preconditioning? PLoS One. 2013;8(12):e83173e83173. doi: 10.1371/journal.pone.0083173 24349453 PMC3862721

[37] Goder R , Aldenhoff JB, Boigs M, Braun S, Koch J, Fritzer G. Delta power in sleep in relation to neuropsychological performance in healthy subjects and schizophrenia patients. J Neuropsychiatry Clin Neurosci. 2006;18(4):529–535. 17135379 10.1176/jnp.2006.18.4.529

[38] D’Rozario AL , Cross NE, Vakulin A, et al. Quantitative electroencephalogram measures in adult obstructive sleep apnea–potential biomarkers of neurobehavioural functioning. Sleep Med Rev. 2017;36:29–42. doi: 10.1016/j.smrv.2016.10.003 28385478

[39] Poon JJ , Chapman JL, Wong KK, et al. Intra-individual stability of NREM sleep quantitative EEG measures in obstructive sleep apnea. J Sleep Res. 2019;28(6):e12838. 30821056 10.1111/jsr.12838

[40] Hoedlmoser K , Heib DP, Roell J, et al. Slow sleep spindle activity, declarative memory, and general cognitive abilities in children. Sleep. 2004;37(9):1501–1512. doi: 10.5665/sleep.4000 PMC4153050 25142558

[41] Carvalho DZ , Gerhardt, GJ, Dellagustin, G, et al. Loss of sleep spindle frequency deceleration in Obstructive Sleep Apnea. Clin Neurophysiol. 2014;125(2):306–312. doi: 10.1016/j.clinph.2013.07.005 23899859

[42] Huupponen E , Himanen SL, Hasan J. et al. Automatic analysis of electro-encephalogram sleep spindle frequency throughout the night. Med Biol Eng Comput. 2003;41(6):727–732. doi: 10.1007/BF02349981 14686599

[43] Chowdhuri S , Quan, SF, Almeida, F. et al. An official american thoracic society research statement: impact of mild obstructive sleep apnea in adults. Am J Respir Crit Care Med. 2016;193(9):e37–e54. doi: 10.1164/rccm.201602-0361ST 27128710

[44] Castronovo V , Canessa N, Strambi LF, et al. Brain activation changes before and after PAP treatment in obstructive sleep apnea. Sleep. 2009;32(9):1161–1172. doi: 10.1093/sleep/32.9.1161 19750921 PMC2737574

[45] Demanuele C , Bartsch U, Baran B, et al. Coordination of slow waves with sleep spindles predicts sleep-dependent memory consolidation in schizophrenia. Sleep. 2017;40(1). doi: 10.1093/sleep/zsw013. PMC6084745 28364465

[46] Muehlroth BE , Sander MC, Fandakova Y, et al. Precise slow oscillation-spindle coupling promotes memory consolidation in younger and older adults. Sci Rep. 2019;9(1):1940–1940. doi: 10.1038/s41598-018-36557-z 30760741 PMC6374430

[47] Mölle M , Bergmann TO, Marshall L, Born J. Fast and slow spindles during the sleep slow oscillation: disparate coalescence and engagement in memory processing. Sleep. 2011;34(10):1411–1421. doi: 10.5665/SLEEP.1290 21966073 PMC3174843

[48] Beaudin AE , Raneri JK, Ayas NT, et al. Cognitive function in a sleep clinic cohort of patients with obstructive sleep apnea. Ann Am ThoracSoc. 2021;18(5):865–875. doi: 10.1513/AnnalsATS.202004-313OC 33147067

